# Functional Outcomes and Recurrence After Arthroscopy-Based Treatment of Pigmented Villonodular Synovitis of the Knee: A 20-Year Single-Center Series

**DOI:** 10.3390/cancers18071178

**Published:** 2026-04-07

**Authors:** Efstathios Konstantinou, Alexandros Koskiniotis, Antonios Koutalos, Konstantinos Malizos, Theofilos Karachalios, Michael Hantes

**Affiliations:** Department of Orthopaedic Surgery and Musculoskeletal Trauma, Faculty of Medicine, School of Health Sciences, University of Thessaly, 41223 Larissa, Greece; efstathios.konstantinou@gmail.com (E.K.); akoskiniot@hotmail.com (A.K.); akoutmed@gmail.com (A.K.); kmalizos@otenet.gr (K.M.); kar@med.uth.gr (T.K.)

**Keywords:** pigmented villonodular synovitis, tenosynovial giant cell tumor, arthroscopic synovectomy, knee joint, disease recurrence

## Abstract

**Background/Objectives**: Tenosynovial giant cell tumor (TGCT), historically referred to as pigmented villonodular synovitis (PVNS), is a rare proliferative synovial disorder that most commonly affects the knee and may lead to progressive joint damage. Surgical synovectomy remains the mainstay of treatment, although recurrence remains a concern, particularly in diffuse disease. The purpose of this study was to evaluate functional outcomes and recurrence following arthroscopic synovectomy with selective open excision for TGCT of the knee. **Methods**: This retrospective case series included patients treated surgically for TGCT of the knee at a single tertiary orthopaedic center between 2003 and 2023. The extended study period may have introduced variability in surgical technique and perioperative management due to evolving clinical practice. All patients underwent arthroscopic synovectomy, with open excision performed when necessary to address posterior or extra-articular disease not amenable to complete arthroscopic resection. Functional outcomes and knee range of motion were evaluated preoperatively and at final follow-up. Recurrence and complications were recorded. **Results**: A total of 43 patients were included with a mean follow-up of 8.1 years. Diffuse TGCT was present in 32 patients (74%), while 11 patients (26%) had localized disease. Arthroscopic synovectomy was performed in all cases, with additional open excision required in 10 patients. Functional scores and range of motion improved significantly at final follow-up (*p* < 0.001). Disease recurrence occurred in 8 patients (18.6%), all with diffuse disease, and was treated with revision arthroscopic synovectomy. **Conclusions**: Arthroscopic synovectomy with selective open excision when required was associated with significant improvement in functional outcomes and knee range of motion. Disease recurrence occurred in 18.6% of patients and exclusively in those with diffuse disease, emphasizing the need for careful postoperative surveillance.

## Simple Summary

Tenosynovial giant cell tumor is a rare condition that affects the lining of joints, most commonly the knee, and can lead to pain, swelling, and progressive joint damage. Surgery is the main treatment, but the disease may return, especially in more extensive forms. In this study, we evaluated the long-term outcomes of patients treated surgically using a minimally invasive arthroscopic technique, with additional open surgery when needed to remove all diseased tissue. We found that patients experienced significant improvement in knee function and movement after surgery. However, recurrence occurred in a proportion of patients, particularly in those with more extensive disease. These findings provide useful information for clinicians in selecting appropriate treatment strategies and highlight the importance of long-term follow-up in managing this condition.

## 1. Introduction

Tenosynovial giant cell tumor (TGCT), historically referred to as pigmented villonodular synovitis (PVNS), is a rare proliferative synovial neoplasm characterized by locally aggressive behavior despite its generally benign histological appearance [1]. Contemporary classifications group these lesions under the broader TGCT terminology and distinguish between localized and diffuse forms, the latter corresponding to classical intra-articular PVNS that frequently involves extensive synovial proliferation and progressive joint damage [2,3]. Although histologically benign, diffuse TGCT may demonstrate aggressive local growth and lead to cartilage degeneration, bone erosion, and functional impairment if not adequately treated [4].

TGCT is characterized by overexpression of colony-stimulating factor 1 (CSF1), typically driven by a chromosomal translocation involving the CSF1 gene. This results in recruitment and accumulation of macrophages expressing the CSF1 receptor, which constitute the majority of the tumor mass and contribute to synovial proliferation and local tissue destruction. This molecular mechanism underlies the characteristic histological appearance of TGCT and has also provided the basis for the development of targeted systemic therapies [1,4].

TGCT is an uncommon condition with an estimated annual incidence of approximately 1.8 cases per million individuals and most commonly affects adults between the third and fifth decades of life [5]. The knee represents the most frequently involved joint and accounts for the majority of reported cases [6]. Patients typically present with nonspecific symptoms including joint pain, swelling, recurrent effusion, and progressive limitation of range of motion, which often results in delayed diagnosis due to overlap with other intra-articular disorders [7]. Magnetic resonance imaging (MRI) is considered the imaging modality of choice because of its high sensitivity for detecting hemosiderin deposition and synovial proliferation, while histopathological examination remains necessary for definitive diagnosis [8].

Surgical synovectomy remains the cornerstone of treatment for TGCT of the knee. Both arthroscopic and open techniques have been described, and combined approaches may be required to achieve complete resection of pathological synovial tissue [9]. Nevertheless, recurrence remains a major challenge, particularly in diffuse disease, with reported recurrence rates varying widely in the literature depending on disease extent and surgical technique [10]. Achieving adequate disease control while minimizing surgical morbidity therefore remains an important objective in the management of this condition [11,12].

In addition to surgical management, alternative and adjunctive therapeutic approaches have been explored, particularly in patients with diffuse or recurrent disease. Adjuvant radiotherapy has been used in selected cases to improve local disease control following synovectomy, although its routine use remains debated given the benign nature of the disease and potential long-term complications [9,10]. Radiosynoviorthesis has also been described as an adjunctive modality aimed at controlling residual synovial proliferation [10]. Furthermore, advances in the understanding of TGCT pathogenesis have led to the development of targeted systemic therapies, particularly inhibitors of the CSF1 receptor, which have shown promising results in patients with unresectable or recurrent disease [1,4].

Despite advances in surgical techniques, optimal management of TGCT of the knee remains debated, particularly regarding the most effective approach to minimize recurrence while preserving joint function [9,10,12]. The purpose of the present study was to evaluate functional outcomes, complication rates, and recurrence following surgical treatment of TGCT of the knee using a standardized approach based on arthroscopic synovectomy with selective open excision when necessary to achieve complete removal of pathological tissue. This study reports long-term outcomes from a relatively large single-center cohort treated with a consistent arthroscopy-based strategy. We hypothesized that arthroscopic synovectomy, combined with open excision when necessary to address posterior and/or extra-articular disease, would result in significant functional improvement with a low recurrence rate at mid- to long-term follow-up.

## 2. Methods

### 2.1. Study Design

This retrospective case series included patients treated surgically for TGCT of the knee at a single tertiary orthopaedic center. Institutional Review Board approval was obtained prior to initiation of the study (approval number: 54235/11-12-25). Patient consent was waived due to the retrospective nature of the study and the use of anonymized clinical data. Patients treated between 2003 and 2023 were identified through institutional medical records and operative databases, resulting in a cohort of 43 patients who met the inclusion criteria. All procedures were performed by experienced senior orthopaedic surgeons at the institution.

### 2.2. Patient Selection

Patients with a confirmed diagnosis of TGCT of the knee who underwent surgical treatment during the study period were eligible for inclusion. The diagnosis was initially suspected based on clinical presentation and imaging findings and was subsequently confirmed by histopathological examination following synovectomy. A minimum follow-up of 2 years was required for inclusion. Patients with incomplete clinical records or insufficient follow-up were excluded from the analysis. A total of 48 patients were initially identified, of whom 5 were excluded due to insufficient follow-up, resulting in a final cohort of 43 patients.

### 2.3. Diagnosis and Preoperative Evaluation

All patients underwent a standardized preoperative clinical evaluation. Magnetic resonance imaging (MRI) of the affected knee was performed preoperatively to assess the extent of synovial involvement and support the diagnosis of TGCT [8]. The final diagnosis was confirmed histologically following surgical excision of the pathological synovial tissue.

### 2.4. Surgical Technique

Arthroscopic synovectomy was performed in all patients as the primary surgical procedure, with the patient in the supine position, including debridement of the anterior and posterior compartments using standard anterolateral and anteromedial portals, as well as posteromedial and posterolateral portals when required. A systematic compartment-by-compartment approach was applied with the aim of complete macroscopic removal of pathological synovial tissue. The posterior compartment was addressed arthroscopically when technically feasible. In cases where complete resection was not achievable arthroscopically, particularly in the presence of limited accessibility or extra-articular extension, a limited open approach was performed to address residual disease, with the specific approach determined by the location and extent of involvement. The surgical objective in all cases was complete removal of visible synovial disease in order to minimize the risk of recurrence.

Postoperatively, early range of motion was encouraged, with weight-bearing as tolerated. Rehabilitation focused on restoring joint mobility and muscle strength, with no standardized use of adjuvant therapies.

### 2.5. Outcome Measures and Follow-Up

Patients were evaluated preoperatively and at final follow-up using validated functional outcome measures, including the Lysholm Knee Score [13], the International Knee Documentation Committee (IKDC) score [14], and the Western Ontario and McMaster Universities Osteoarthritis Index (WOMAC) [15]. Range of motion (ROM) of the knee was also recorded.

Postoperative complications were documented. Disease recurrence was assessed through clinical evaluation during follow-up visits and with MRI when clinically indicated. Recurrence was defined as the presence of recurrent symptoms associated with imaging findings consistent with TGCT and/or the need for revision surgical treatment.

### 2.6. Statistical Analysis

Descriptive statistics were calculated for all variables. Continuous variables were reported as mean values with ranges, while categorical variables were presented as frequencies and percentages. Differences between preoperative and postoperative functional outcomes, including the Lysholm score, IKDC score, WOMAC score, and knee ROM, were assessed using paired comparisons. A *p*-value < 0.05 was considered to indicate statistical significance. All statistical analyses were performed using standard statistical software. Paired *t*-tests were used to evaluate pre- and postoperative changes.

Artificial intelligence-assisted language tools were used for minor language editing and formatting of the manuscript. All scientific content, analysis, and interpretation were performed and verified by the authors.

## 3. Results

A total of 43 patients with TGCT of the knee were included in the study. The cohort consisted of 21 male and 22 female patients with a mean age of 30.6 years (range, 8–65 years) at the time of surgery. Four patients were pediatric or adolescent (one aged 8 years, one aged 15 years, and two aged 17 years). No differences in treatment, follow-up, or outcome assessment were applied between these patients and the adult cohort. The mean clinical follow-up was 8.1 ± 2.8 years.

Right knee involvement was observed in 24 patients and left in 19. Extra-articular extension was present in four patients. Among patients with diffuse disease, open excision was more frequently required compared to localized disease. No patients had a history of prior surgical treatment for TGCT.

Diffuse TGCT was identified in 32 patients (74%), whereas 11 patients (26%) presented with localized disease. Arthroscopic synovectomy was performed in all cases. In 10 patients, an additional open excision was required in order to achieve complete removal of pathological tissue, most commonly due to posterior compartment involvement not fully accessible arthroscopically or extra-articular extension.

Functional outcomes improved significantly following surgical treatment. The Lysholm score, IKDC score, and WOMAC score all demonstrated statistically significant improvement at final follow-up compared with preoperative values. Knee ROM also improved significantly after surgery (*p* < 0.001 for all comparisons). The overall direction of change for these outcome measures is summarized in Table 1, and reflects values at final follow-up, including patients who underwent revision procedures.

Disease recurrence occurred in 8 patients (18.6%) during the follow-up period. All recurrences were observed in patients with diffuse TGCT, corresponding to a recurrence rate of 25.0% within the diffuse subgroup, while no recurrences occurred in patients with localized disease (Table 2). The mean time to recurrence following the index procedure was 3.8 ± 1.9 years. All recurrences were treated with revision arthroscopic synovectomy.

No major postoperative complications, defined as complications requiring reoperation or resulting in significant morbidity, were recorded during the study period. Minor postoperative complications included prolonged joint stiffness in nine patients, which was successfully managed with physiotherapy. No cases of infection or thromboembolic events were observed.

## 4. Discussion

The most important finding of the present study is that surgical treatment of TGCT of the knee using an arthroscopy-based approach with selective open excision was associated with significant functional improvement and a recurrence rate of 18.6% at long-term follow-up. In this cohort of 43 patients with a mean follow-up of 8.1 years, all functional outcome measures improved significantly following surgery, while recurrence occurred in 18.6% of cases and exclusively in patients with diffuse disease. These findings support the role of complete synovectomy as the cornerstone of treatment for TGCT of the knee and are consistent with outcomes reported in previous surgical series [10,11,12].

Pigmented villonodular synovitis, currently classified within the spectrum of tenosynovial giant cell tumors, is a rare proliferative synovial disorder that most frequently affects large joints, particularly the knee [5,6]. The disease is typically classified into localized and diffuse forms, with the diffuse variant generally demonstrating a more aggressive clinical course and a higher propensity for recurrence [3,9]. In the present series, diffuse TGCT accounted for 74% of cases, while localized disease was observed in 26%, reflecting the predominance of diffuse disease reported in previous surgical cohorts [3,5]. This distribution likely reflects the greater clinical impact of diffuse disease, which more frequently necessitates surgical intervention compared with localized nodular lesions.

Surgical synovectomy remains the primary treatment for TGCT of the knee, although the optimal surgical approach continues to be debated. Both open and arthroscopic synovectomy have been described, and some authors advocate combined techniques in order to achieve more complete removal of diseased synovial tissue, particularly in cases of diffuse disease [9,12,16]. Arthroscopic synovectomy offers several potential advantages, including improved visualization of intra-articular compartments, reduced surgical morbidity, and faster postoperative recovery, and has therefore gained increasing popularity in the management of TGCT of the knee [10,17]. Nevertheless, open procedures may still be required in selected cases, particularly when posterior compartment involvement or extra-articular extension limits the ability to achieve complete resection arthroscopically [11]. In the present study, all patients were initially treated arthroscopically, with selective open excision performed when complete removal of pathological tissue could not be achieved through arthroscopy alone.

Recurrence remains one of the principal challenges in the management of TGCT of the knee and represents a key determinant of long-term outcomes [18]. In the present study, the overall recurrence rate was 18.6%, which falls within the range reported in previous surgical series. Reported recurrence rates vary widely in the literature, largely depending on disease type and surgical technique, with values ranging from approximately 10% to more than 40%, particularly in cases of diffuse TGCT [9,10,11]. In the current cohort, all recurrences occurred in patients with diffuse disease, corresponding to a recurrence rate of 25% within this subgroup, while no recurrences were observed in patients with localized TGCT. This finding is consistent with previous reports demonstrating that diffuse TGCT carries a substantially higher risk of recurrence due to the more extensive synovial involvement and the difficulty of achieving complete resection [5,12]. The mean time to recurrence of 3.8 years observed in the present series is also comparable to previous studies reporting that most recurrences occur within the first several years following surgical treatment [11]. This finding highlights that recurrence may occur several years after the index procedure, particularly in patients with diffuse disease, and supports the need for continued long-term clinical surveillance.

Functional outcomes also improved substantially following surgical treatment in the present cohort. As shown in Table 1, the mean Lysholm score improved from 47 ± 15 preoperatively to 88 ± 12 at final follow-up, while the IKDC score increased from 42 ± 14 to 81 ± 13. Similarly, the WOMAC score improved from 51 ± 18 to 19 ± 10, reflecting a marked reduction in symptoms and functional limitations. Knee ROM also improved from 112° ± 15 preoperatively to 131° ± 8 at final follow-up. These findings are comparable to previously reported surgical series, which have demonstrated meaningful improvements in knee function and patient-reported outcome measures following synovectomy for TGCT of the knee [10,11,12,19]. Nevertheless, complete restoration of knee function is not always achieved, particularly in patients with diffuse disease or long-standing synovial involvement, which may be associated with persistent symptoms or functional limitations despite surgical treatment.

In addition to surgical management, several adjuvant and systemic treatment strategies have been described for TGCT, particularly in cases of diffuse or recurrent disease. Adjuvant radiotherapy has been proposed as a means of reducing recurrence after synovectomy in selected patients with extensive synovial involvement, although its routine use remains controversial due to concerns regarding potential complications and the benign nature of the disease [9,20]. Radiosynoviorthesis has also been explored as an adjunct treatment aimed at controlling residual synovial disease following surgery [20]. More recently, advances in the understanding of the molecular pathogenesis of TGCTs have led to the development of targeted systemic therapies, particularly inhibitors of the CSF1 receptor [21]. Agents such as pexidartinib have demonstrated clinical activity in patients with advanced or unresectable disease and represent an emerging therapeutic option in selected cases [1,21]. These treatment modalities are generally reserved for patients with unresectable, recurrent, or extensive diffuse disease, particularly when complete surgical resection is not feasible or is associated with high morbidity. In the present cohort, however, all patients were managed surgically, and adjuvant radiotherapy or systemic therapies were not utilized due to limited availability during the study period.

The etiology of TGCT remains incompletely understood, and no definitive risk factors have been consistently identified, although prior trauma has been suggested as a possible contributing factor in some cases [1,5]. While screening strategies are not applicable to this rare condition, early diagnosis remains clinically important, as delayed recognition may allow disease progression with more extensive synovial involvement and subsequent cartilage damage, potentially leading to worse functional outcomes [3,5].

Although arthroscopic synovectomy is widely used in the management of TGCT of the knee, achieving complete removal of pathological synovial tissue remains challenging, particularly in cases of diffuse disease involving the posterior compartment or extending beyond the joint capsule. Incomplete resection has been associated with an increased risk of recurrence, highlighting the importance of a systematic and thorough surgical approach [9,10,11]. In this context, combining arthroscopic techniques with selective open excision may allow more complete disease removal while maintaining the advantages of minimally invasive surgery [10,12]. However, the balance between surgical completeness and procedure-related morbidity remains an important consideration, especially in patients with extensive disease involvement.

Despite the findings presented in this study, several limitations should be acknowledged. First, the retrospective design inherently introduces potential sources of bias, including variability in clinical documentation and follow-up evaluation. Second, this study represents the experience of a single tertiary center, which may limit the generalizability of the findings to other institutions with different patient populations or treatment strategies. In addition, surgical treatment was performed by multiple surgeons over a long study period, which may have introduced some variability in surgical technique and perioperative management. The extended study period also encompasses potential changes in MRI quality, evolution of arthroscopic instrumentation and surgical techniques, as well as variations in surgical experience, in addition to shifts in pathological classification and postoperative surveillance practices, which may have influenced the diagnosis, treatment, and follow-up of patients over time. These factors may have affected the detection of disease extent, surgical decision-making, and the identification of recurrence, thereby introducing heterogeneity in the reported outcomes. Furthermore, as MRI was performed only when clinically indicated, asymptomatic recurrence may have been underdiagnosed. Finally, although the present series provides long-term follow-up, the relatively small number of patients reflects the rarity of the disease and limits the ability to perform more detailed subgroup analyses. Nevertheless, the present study also has notable strengths, including a relatively large cohort for this rare condition and a long mean follow-up of more than eight years, which allowed meaningful evaluation of recurrence patterns following surgical treatment.

## 5. Conclusions

Arthroscopic synovectomy with selective open excision when required was associated with significant improvement in functional outcomes and knee range of motion in patients with TGCT of the knee at long-term follow-up. Recurrence occurred in 18.6% of patients and exclusively in those with diffuse disease, highlighting the more aggressive nature of this subtype and the need for careful postoperative surveillance. These findings contribute to the understanding of long-term outcomes and recurrence patterns following arthroscopy-based management of TGCT of the knee.

## Figures and Tables

**Table 1:** Functional outcomes before surgery and at final follow-up.

Outcome Measure	Preoperative Mean ± SD	Final Follow-Up Mean ± SD	*p*-Value
Lysholm score	47 ± 15	88 ± 12	<0.001
IKDC score	42 ± 14	81 ± 13	<0.001
WOMAC score	51 ± 18	19 ± 10	<0.001
Knee ROM (°)	112 ± 15	131 ± 8	<0.001

**Table 2:** Distribution of disease recurrence according to TGCT subtype.

Disease Type	Number of Patients	Recurrences	Recurrence Rate
Localized TGCT	11	0	0%
Diffuse TGCT	32	8	25.0%
Total	43	8	18.6%

## Data Availability

The data supporting the findings of this study are available from the corresponding author upon reasonable request.

## Abbreviations

CSF1	Colony-Stimulating Factor 1
IKDC	International Knee Documentation Committee
MRI	Magnetic Resonance Imaging
PVNS	Pigmented Villonodular Synovitis
ROM	Range of Motion
TGCT	Tenosynovial Giant Cell Tumor
WOMAC	Western Ontario and McMaster Universities Osteoarthritis Index
